# Prevalence of plasmid-bearing and plasmid-free *Chlamydia trachomatis* infection among women who visited obstetrics and gynecology clinics in Malaysia

**DOI:** 10.1186/s12866-016-0671-1

**Published:** 2016-03-18

**Authors:** Tee Cian Yeow, Won Fen Wong, Negar Shafiei Sabet, Sofiah Sulaiman, Fatemeh Shahhosseini, Grace Min Yi Tan, Elaheh Movahed, Chung Yeng Looi, Esaki M. Shankar, Rishien Gupta, Bernard P. Arulanandam, Jamiyah Hassan, Sazaly Abu Bakar

**Affiliations:** Department of Medical Microbiology, Tropical Infectious Disease Research and Education Center, Faculty of Medicine, University of Malaya, 50603 Kuala Lumpur, Malaysia; Faculty of Medicine, SEGi University, 47810 Petaling Jaya, Malaysia; Department of Obstetrics and Gynecology, Faculty of medicine, University of Malaya, 50603 Kuala Lumpur, Malaysia; Department of Pharmacology, Faculty of Medicine, University of Malaya, 50603 Kuala Lumpur, Malaysia; Center of Excellence in Infection Genomics, South Texas Center For Emerging Infectious Diseases, University of Texas at San Antonio, 78249 San Antonio, TX USA

**Keywords:** *Chlamydia trachomatis*, Reproductive system disorders, Infertility, Plasmid

## Abstract

**Background:**

The 7.5 kb cryptic plasmid of *Chlamydia trachomatis* has been shown to be a virulence factor in animal models, but its significance in humans still remains unknown. The aim of this study was to investigate the prevalence and potential involvement of the *C. trachomatis* cryptic plasmid in causing various clinical manifestations; including infertility, reproductive tract disintegrity, menstrual disorder, and polycystic ovarian syndrome (PCOS) among genital *C. trachomatis*–infected patients.

**Results:**

A total of 180 female patients of child bearing age (mean 30.9 years old, IQR:27–35) with gynecological complications and subfertility issues, who visited Obstetrics and Gynecology clinics in Kuala Lumpur, Malaysia were recruited for the study. Prevalence of genital chlamydial infection among these patients was alarmingly high at 51.1 % (92/180). Of the 92 chlamydia-infected patients, 93.5 % (86/92) were infected with plasmid-bearing (+) *C. trachomatis* while the remaining 6.5 % (6/92) were caused by the plasmid-free (−) variant. Our data showed that genital *C. trachomatis* infection was associated with infertility issues, inflammation in the reproductive tract (mucopurulent cervicitis or endometriosis), irregular menstrual cycles and polycystic ovarian syndrome (PCOS). However, no statistical significance was detected among patients with plasmid (+) versus plasmid (−) *C. trachomatis* infection. Interestingly, plasmid (+) *C. trachomatis* was detected in all patients with PCOS, and the plasmid copy numbers were significantly higher among PCOS patients, relative to non-PCOS patients.

**Conclusion:**

Our findings show a high incidence of *C. trachomatis* infection among women with infertility or gynecological problems in Malaysia. However, due to the low number of plasmid (−) *C. trachomatis* cases, a significant role of the plasmid in causing virulence in human requires further investigation of a larger cohort.

## Background

*Chlamydia trachomatis* is a common bacterial sexually transmitted disease (STD) worldwide. Chlamydial urogenital infection has increased at a fast rate, with more than 2.8 million new cases diagnosed each year [1]. In Malaysia, a high prevalence of chlamydial antibody was detected among urban citizens [2], particularly young females and sex workers [3]. Long term asymptomatic persistency of the pathogen among 50–70 % of individuals results in wide spread of the disease [4], and leads to delayed treatment, which in turn causes prostatitis and epididymitis in males or infertility in females [5]. In females, the pathogen can ascend the reproductive tract to the endometrial epithelium and fallopian tubes, leading to pelvic inflammatory disease (PID) in approximately 20–40 % of the infected patients; among which 11 % develop tubal factor infertility while 9 % show ectopic pregnancy [6]. *C. trachomatis*-mediated inflammation in the genital tract can result in cervicitis or endometriosis, causing lesions, abnormal mucopurulent discharge and thickening of the uterus inner layer [6]. Inflammation can also contribute to hormonal disorders, i.e., polycystic ovarian syndrome (PCOS) and irregular menses [7]. Additionally, newborn infants can be infected at birth when delivered through an infected birth canal, causing severe conjunctivitis or pneumonia [8].

**Fig. 1:**
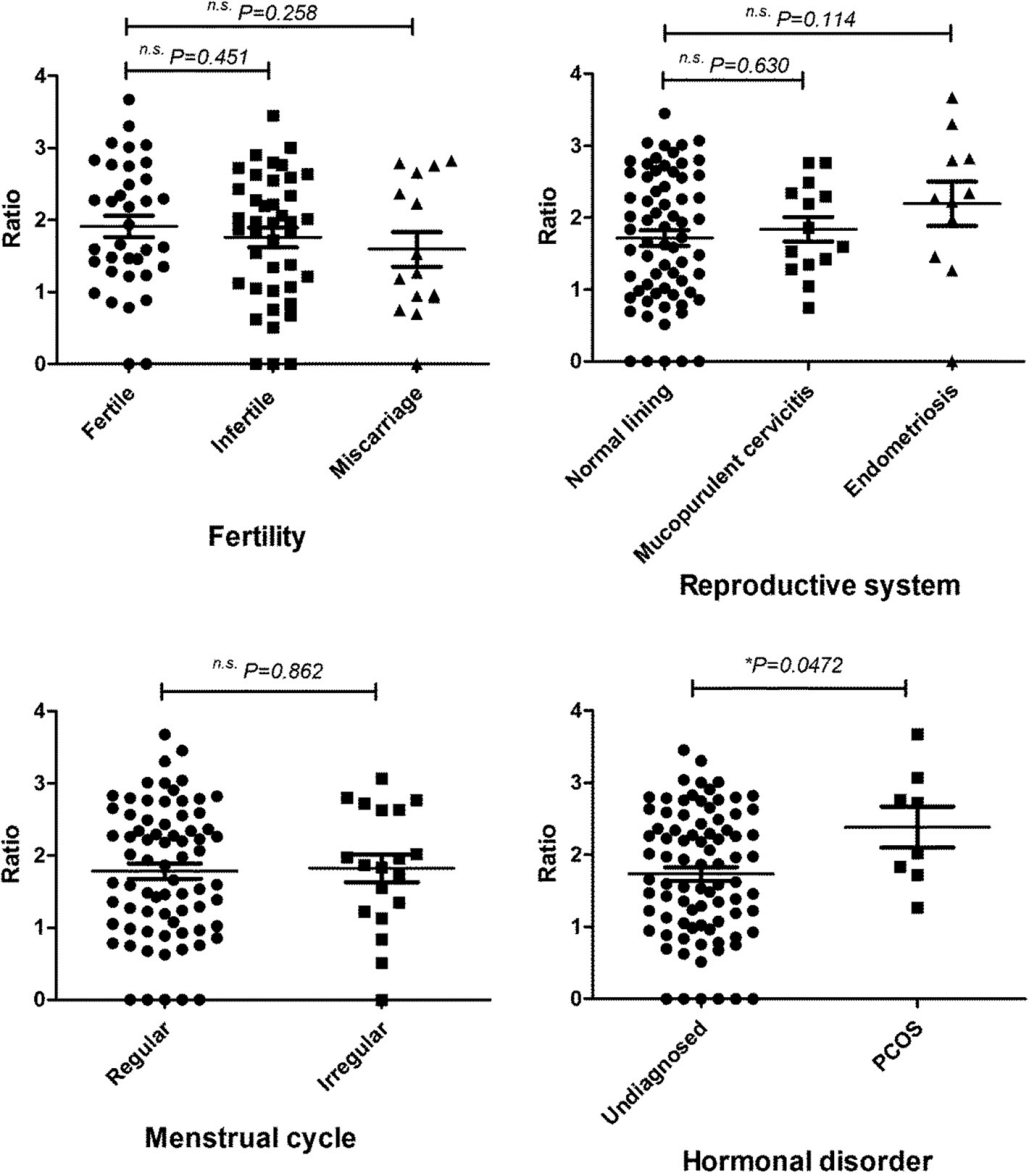
Plasmid copy numbers among *C. trachomatis*-infected patients with different clinical parameters. Dot plot graphs show the ratio of plasmid:*Momp* as determined by quantitative real-time PCR assay. Each dot represents data from a single patient in each group (*n* = 92). The *P* values were measured with unpaired Student’s *t*-test. Data was considered significant when *P < 0.05. *n.s.*: non-significant

In recent years, several research groups suggest that the *C. trachomatis* without plasmid demonstrates a weaker degree of virulence. The plasmid-deficient strains show impaired ability to infect the female mouse genital tract [9, 10]. *C. trachomatis* infection damages the reproductive tract by activating TLR2 and initiates a Th1-dependent inflammatory response. In a study using plasmid-cured *C. muridarum* mutant infection in a murine model, TLR2-dependent cytokine was not detected and no sign of oviduct damage can be observed, suggesting that the plasmid-cured mutant has lost virulence [9]. Also, intra-vaginal immunization of the plasmid-deficient strains provide protection in the murine model of genitourinary tract infection [11]; as well as in trachoma infected macaques [12] due to the failure to trigger a Toll-like receptor 2-dependent immune response [13]. These findings have led to research on use of plasmid-deficient strains as a potential human vaccine. In fact, an intravaginal infection of attenuated plasmidless *C. trachomatis* L2 has been shown to provide protection to the host because it is nonpathogenic and raises systemic antibody in the C3H/HeJ mouse model [11].

Despite compelling evidence in animal models, there is no equivalent finding from human clinical study confirming the significance of plasmid in *C. trachomatis* infection. A recent study in human ocular *C. trachomatis* infection reveals that no direct association exists between plasmid copy numbers and disease severity [14]. Therefore, further work should be done to establish the plausible connection. In this study, we investigated the prevalence of plasmid-bearing (+) or plasmid-free (−) *C. trachomatis* infection and the plasmid copy numbers among obstetrics and gynecology patients in Kuala Lumpur, the capital city of Malaysia. By associating the data with the patients’ clinical presentations, we evaluated the prevalence and risk of plasmid (+) and plasmid (−) variants in affecting gynecological disorders.

## Results

### Demographics of the study population

The patient cohort from this study comprised 180 female patients of child-bearing age (mean: 30.95 years old; IQR: 27–35) who visited the Obstetrics and Gynecology clinic at the University of Malaya Medical Centerfrom 2010 to 2014 (Table 1). A total number of 124 patients were married, 13 were single or had divorced, and the remaining 43 patients did not reveal their marital status. Most of the patients were of Malay ethnicity (68.3 %), followed by Indians (13.3 %), Chinese (12.7 %), and others (5.5 %), reflecting the demographics of the multiethnic Malaysian population. A majority of the patients recruited to the study presented with gynecological complications or subfertility issues, and had a higher risk of STD infection. The main reason for patients’ visit to the clinic was due to infertility (37 %). Other reasons included abdominal pain, bleeding, menorrhagia, irregular menstrual cycles and others. Two patients had cystectomy whereas one had a history of resolved PID.

**Table 1:** Patient demographics. Numbers (percentages) of patients with or without *C. trachomatis* infection; and patients infected with plasmid (−) or plasmid (+) variants. *n* = 180. The *P* values for all variables were measured with Fisher’s exact test. For age, a *t*-test (^a^) was used. *n.s.*: non-significant

Parameters	All patients (*n* = 180)	No infection (*n* = 88)	*C. trachomatis* infection (*n* = 92)	*P* value	OR (95 % CI)	Plasmid (−) *C. trachomatis* (*n* = 6)	Plasmid (+) *C. trachomatis* (*n* = 86)	*P* value	OR (95 % CI)
Age (years)									
Mean [IQR]	30.9 [27–35]	30.7 [27–34.25]	31.29 [27–35]	0.60 ^*n.s.*^ ^a^		30.2 [26.75–32.25]	33.3 [27–35]	0.59 ^*n.s.*^ ^a^	
Maximum	45	44	45			36	45		
Minimum	20	20	20			26	20		
Marital status									
Married	124 (68.8 %)	55 (44.4 %)	69 (55.6 %)	0.078 ^*n.s.*^	1.800 (0.94–3.41)	3 (4.3 %)	66 (95.6 %)	0.16 ^*n.s.*^	0.303 (0.056–1.62)
Single	12 (6.7 %)	6 (50 %)	6 (50 %)	1.00 ^*n.s.*^	0.953 (0.29–3.07)	0 (0 %)	6 (100 %)	1.00 ^*n.s.*^	0.952 (0.048–18.87)
Divorced	1 (0.5 %)	0 (0 %)	1 (100 %)	1.00 ^*n.s.*^	2.902 (0.11–72.24)	0 (0 %)	1 (100 %)	1.00 ^*n.s.*^	4.385 (0.16–118.8)
unknown	43 (23.8 %)	27 (62.7 %)	16 (37.3 %)	0.053 ^*n.s.*^	0.475 (0.23–0.96)	3 (18.7 %)	13 (81.2 %)	0.60 ^*n.s.*^	5.615 (1.02–3.09)
Ethnicity									
Malay	123 (68.3 %)	64 (52.1 %)	59 (47.9 %)	0.26 ^*n.s.*^	0.6705 (0.35–1.26)	4 (6.7 %)	55 (93.2 %)	1.00 ^*n.s.*^	1.127 (0.19–6.51)
Indian	24 (13.3 %)	11 (45.9 %)	13 (54.1 %)	0.82 ^*n.s.*^	1.152 (0.48–2.72)	1 (7.6 %)	12 (92.3 %)	1.00 ^*n.s.*^	1.233 (0.13–11.50)
Chinese	23 (12.7 %)	10 (43.5 %)	13 (56.5 %)	0.65 ^*n.s.*^	1.284 (0.53–3.10)	1 (7.6 %)	12 (92.3 %)	1.00 ^*n.s.*^	1.233 (0.13–11.50)
Others	10 (5.5 %)	3 (30 %)	7 (70 %)	0.33 ^*n.s.*^	2.333 (0.58 to 9.32)	0 (0.0 %)	7 (100.0 %)	1.00 ^*n.s.*^	0.815 (0.13–11.50)

### *C. trachomatis* infection is associated with various gynecological disorders

Genital *C. trachomatis* infection in each patient was first diagnosed by detection of the *C. trachomatis Momp* (*OmpA*) gene. Total DNA was first isolated from the patients’ endocervical swabs and PCR amplified using a human β-globin control primer to ensure the successful extraction of DNA from the endocervical swabs (data not shown). The presence of *C. trachomatis* DNA was detected with *Momp* primer using a nested PCR amplification method (outer primers followed by inner primer amplification) [15]. The results were further confirmed using a quantitative real-time PCR method with inner primer alone. MOMP is expressed on the cell surface with a porin activity that is often used for diagnosis of *C. trachomatis* [16]. Over half of the patients, i.e., 51.1 % (92/180), were diagnosed with genital *C. trachomatis* infection, suggesting a high incidence of sexually transmitted chlamydial infection among the female patients recruited (Table 2). No significant difference was detected among patients by age, marital status or ethnic background. We then sequenced the *Momp* PCR product and performed a nucleotide BLAST to assess the diversity of *C. trachomatis* strains in the local population. Interestingly, our data revealed that all samples examined showed a D serotype (data not shown).

**Table 2:** Chlamydial infection in patients with different symptoms. Numbers (percentages) of patients with or without *C. trachomatis* infection. *n* = 180. The *P* values for all variables were measured with Fisher’s exact test*. n.s.*: non-significant

Parameters	All patients (*n* = 180)	Non-infected (*n* = 88)	*C. trachomatis* -infected (*n* = 92)	*P* value	Odd Ratio (95 % CI)
Fertility					
Fertile	112	76 (67.9 %)	36 (32.1 %)		
Infertile	68	12 (17.6 %)	56 (82.4 %)	<0.0001^***^	9.852 (4.70 to 20.63)
- 1° or 2° infertility	43	4 (9.3 %)	39 (90.7 %)	< 0.0001^***^	20.580 (6.83 to 62.02)
- Miscarriage	25	8 (32.0 %)	17 (68.0 %)	0.0013^**^	4.486 (1.77 to 11.36)
Reproductive tract					
Normal lining	143	79 (55.2 %)	64 (44.8 %)		
Inflammation	37	9 (24.3 %)	28 (75.7 %)	0.00088^***^	3.84 (1.69 to 8.72)
- Mucopurulent Cervicitis	25	9 (36.0 %)	16 (64.0 %)	0.0876 ^*n.s.*^	2.167 (0.90 to 5.23)
- Endometriosis	12	0 (0.0 %)	12 (100.0 %)	0.0001^***^	30.43 (1.77 to 524.2)
Menstrual Cycle					
Regular	130	70 (53.8 %)	60 (46.2 %)		
Irregular	50	18 (36.0 %)	32 (64.0 %)	0.0452^*^	2.074 (1.05 to 4.06)
Hormonal Disorder					
Undiagnosed	170	87 (51.2 %)	83 (48.8 %)		
PCOS	10	1 (10.0 %)	9 (90.0 %)	0.0184^*^	9.434 (1.17 to 76.14)

*P<0.05 **P<0.01 ***P<0.001

Overall, *C. trachomatis* infection showed an association with infertility (OR:9.85, 95 % CI:4.70–20.63, *P* < 0.0001^***^) (Table 2). Among patients who were fertile, only 32.1 % (36/112) were infected by *C. trachomatis*, whereas among infertile patients, 82.4 % (56/68) had *C. trachomatis* infection. Notably, a high *C. trachomatis* prevalence of 90.7 % (39/43,) was detected among patients who were diagnosed with primary (1°) or secondary (2°) infertility (OR:20.580, 95 % CI:6.83–62.02, *P* < 0.0001^***^). Meanwhile, 68 % (17/25) of patients who experienced miscarriage were infected by *C. trachomatis* (OR:4.486, 95 % CI:1.77–11.36, *P* = 0.0013^**^).

The inflammation (cervicitis with mucopurulent discharge or endometriosis) of patients’ reproductive tract was recorded by clinicians during examination. *C. trachomatis* infection showed a close connection with the inflammation in the reproductive system (OR:3.84, 95 % CI:1.69–8.72, *P* = 0.0008^***^) (Table 2). Our data showed that approximately 64 % (16/25) of patients who were diagnosed with mucopurulent cervicitis (OR:2.167, 95 % CI:0.90–5.23, *P* = 0.087) and 100 % (12/12) of patients with endometriosis (OR:30.43, 95 % CI:1.77–524.2, *P* < 0.0001^***^) had *C. trachomatis* infection.

In addition, we showed that *C. trachomatis* infection exhibited significant association with irregular menstrual cycles (OR:2.07, 95 % CI:1.05–4.06, *P* = 0.045^*^) as well as PCOS (OR:9.43, 95 % CI:1.17–76.14, *P* = 0.018^*^), when compared to the uninfected controls.

### Higher incidence of plasmid-bearing *C. trachomatis* infection among patients with infertility, inflammation and PCOS

Next, we investigated if the patients enrolled were infected by plasmid (+) or (−) *C. trachomatis* variants. Total DNA extracted from the vaginal swabs of the *C. trachomatis*-infected patients were amplified with two sets of cryptic plasmid primers (which target *Pgp1* and *Pgp8* respectively) using quantitative real-time PCR analysis. Of the 92 *C. trachomatis-*infected patients, we detected that 93.5 % (86/92) were caused by plasmid (+) *C. trachomatis* whereas only 6.5 % (6/92) were caused by plasmid (−) *C. trachomatis* (Table 1).

When comparing the clinical parameters of the patients, we noted no significant correlation between the plasmid and the tendency for the patients to exhibit infertility (OR:0.76, 95 % CI:0.13–4.40, *P* = 0.562), inflammation (OR:5.26, 95 % CI:0.28–98.68, *P* = 0.155), irregular menses (OR:2.81, 95 % CI:0.31–25.24, *P* = 0.315) and PCOS (OR:1.59, 95 % CI:0.08–30.6, *P* = 0.529) (Table 3). Among the patients with infertility, 92.8 % (52/56) cases were diagnosed with plasmid (+) *C. trachomatis*, compared to only 7.14 % (4/56) cases with plasmid (−) *C. trachomatis*. Similarly, the majority of the patients who had inflammation in the reproductive tract (96.4 %, 27/28) and PCOS (100 %, 9/9) were caused by plasmid (+) *C. trachomatis*. Among the 6 patients infected by plasmid (−) *C. trachomatis*, 2 were fertile while 4 had infertility. However, 5 among the 6 showed no sign of inflammation and had regular menstrual cycles.

**Table 3:** *C. trachomatis* plasmid in patients with different symptoms. Numbers (percentages) of patients infected with plasmid (−) or plasmid (+) *C. trachomatis* variants. *n* = 92. The *P* values for all variables were measured with Fisher’s exact test. *n.s.*: non-significant

Parameters	*C. trachomatis-*infected patients (*n* = 92)	Plasmid (−) *C. trachomatis* (*n* = 6)	Plasmid (+) *C. trachomatis* (*n* = 86)	*P* value	Odd Ratio (95 % CI)
Fertility					
Fertile	36	2 (5.55 %)	34 (94.4 %)		
Infertile	56	4 (7.14 %)	52 (92.8 %)	0.5629 ^*n.s.*^	0.764 (0.13 to 4.40)
- 1° or 2° infertility	39	3 (7.6 %)	36 (92.3 %)	0.5295 ^*n.s.*^	0.755 (0.14 to 3.95)
- Miscarriage	17	1 (5.8 %)	16 (88.2 %)	0.6939 ^*n.s.*^	0.875 (0.09 to 8.01)
Reproductive tract					
Normal lining	64	5 (7.8 %)	59 (92.1 %)		
Inflammation	28	1 (3.5 %)	27 (96.4 %)	0.4045 ^*n.s.*^	2.288 (0.25 to 20.55)
- Mucopurulent Cervicitis	16	0 (0.0 %)	16 (100 %)	0.1550 ^*n.s.*^	5.269 (0.28 to 98.68)
- Endometriosis	12	1 (8.3 %)	11 (91.6 %)	0.5786 ^*n.s.*^	0.733 (0.078 to 6.88)
Menstrual Cycle					
Regular	60	5 (8.3 %)	55 (91.6 %)		
Irregular	32	1 (3.1 %)	31 (96.8 %)	0.3153 ^*n.s.*^	2.818 (0.31 to 25.24)
Hormonal Disorder					
Undiagnosed	83	6 (72.2 %)	77 (92.7 %)		
PCOS	9	0 (0 %)	9 (100 %)	0.5293 ^*n.s.*^	1.59 (0.083 to 30.6)

### Higher relative plasmid copy numbers associated with PCOS

To examine if the abundance of plasmid in *C. trachomatis* is associated with the presence of the clinical symptoms, we quantified the plasmid copy numbers in each patient (Figure 1). The average C_T_ value from two cryptic plasmid primers (*Pgp1* and *Pgp8*) for each patient was obtained. Relative plasmid copy numbers per bacterium (ratio for plasmid:*Momp*) for all 92 *C. trachomatis*-infected patients were calculated. In general, we noticed no direct connection between fertility, the reproductive tract lining and menstrual cycle, consistent with the previous studies in human ocular infection and *in vitro* tissue tropism [14, 17]. Notably, PCOS patients showed a higher plasmid copy number (2.386 ± 0.284 versus 1.734 ± 0.096, *P* = 0.0472), relative to the non-PCOS patients. Although endometriosis patients also demonstrated a comparatively higher plasmid copy number, (2.193 ± 0.308 versus 1.714 ± 0.110, *P* = 0.114), no statistical significance was noted.

## Discussion

In this study, we reported a high prevalence (51.1 %) of *C. trachomatis* infection among female adults of child bearing age, with subfertility or gynecological problems who visited Obstetrics and Gynecology clinics in Malaysia. This indicates that there is a pressing need for wider population screening to increase awareness and prevent the spread of the disease among the community. Most of the patients who demonstrated symptoms were diagnosed with genital *C. trachomatis* infection, including infertility (82.4 %), reproductive system lesions (75.7 %), irregular menses (64 %) and PCOS (90 %), suggesting the *C. trachomatis* is a leading factor for female reproductive system disorders.

Although a high rate of genital *C. trachomatis* prevalance was detected in our study, the patients recruited were suspected to be at risk of the bacterial infection based on the clinical examination. The rates of *C. trachomatis* infection vary in different studies depending on the group of patients recruited and the study region. For example, a study using 50 infertile female patients showed a 40 % infection rate by *C. trachomatis* [18] whereas other studies reported only a 8 % [19] or 15 % *C. trachomatis* infection rate. Among patients with tubal infertility, the prevalence of *C. trachomatis* was reported to be 38.3 % among 120 patients [20]. Consistent to the previous study, the presence of plasmid (+) *C. trachomatis* was high (93.5 %). However, the prevalence using real-time PCR amplification depends highly on the assay sensitivity. For data validation, a glycogen-positive test for bacterial isolates can be used to confirm the presence of plasmid in the bacteria [21].

Various research groups have established that the plasmid (+) *C. trachomatis* demonstrated a higher virulence in animal models of ocular or genital infections [9–14]. Consistent with these findings, our results showed that high percentages of those who had infertility (92.8 %), inflammation in the reproductive tract (96.4 %), irregular menses (96.8 %) and PCOS (100 %) were diagnosed with plasmid (+) but not plasmid (−) variants of *C. trachomatis*. Although these observations provide support for previous work which suggests the usage of plasmidless *C. trachomatis* as a potential human vaccine [11], caution must be taken as some of the patients who showed symptoms were infected by the plasmid (−) strains. Also note that the genetic diversity of human populations may contribute to results contradicting experiments performed using inbred animals. Therefore, additional surveys which involve a larger human cohort should be conducted to ensure the over-all safety of the plasmidless strains among individuals from diverse backgrounds.

Plasmid (+) or (−) *C. trachomatis* strains demonstrated no difference with regards to growth kinetics, plaquing efficiency and size but showed a defect in glycogen granule accumulation and intrainclusion movement [10]. A recent study showed that *C. trachomatis* is capable of inducing alteration to global host histone modifications and double strand break repair, thus generating an environment favorable for malignant transformation [22]. In fact, *C. trachomatis* infection poses risk for infected individuals to develop cervix intraepithelial neoplasia [23, 24], in a similar way to other tumor-inducing pathogens. Extra-chromosomal plasmid DNA may play a role in integrating with the host genome which leads to this pathological damage. The conserved 7.5 kb cryptic plasmid is a small, non-conjugative and non-integrative extrachromosomal DNA that contains genes encoding 8 proteins [25]. The plasmid *Pgp3* encodes for immunogenic trimers which trigger specific antibody production in infected individuals [26, 27]; and is secreted into the cytosol of infected cells during chlamydial infection [28, 29]. Meanwhile, *Pgp4* encodes for a protein which comprises a putative helix-loop-helix domain which functions as transcriptional regulator for virulence-associated genes [10, 30]. Therefore, expression of plasmid genes may be crucial in leading to clinical symptoms. To support this notion, a quantitative protein analysis could be carried out in the future to measure the amount of plasmid-derived proteins in the patient sample.

## Conclusion

In conclusion, we suggest that plasmids maybe a potential risk factor for reproductive system disorders including infertility, inflammation in the reproductive tract, irregular menses and PCOS in genital chlamydial infection. However, continued work is required to substantiate plasmids as a virulence factor in future studies with a larger human cohort.

## Methods

### Study design

A total number of 180 female patients of child-bearing age who visited the Obstetrics and Gynecology Outpatient Clinics at the University of Malaya Medical Centre voluntarily participated in the study from 2010 to 2014. Detailed clinical information on reasons for referral, gynecological history including menstruation, symptoms of genital and urinary tract infection, obstetric and medical histories were documented. The subjects’ vulva and cervix were examined for the presence of lesions, warts, ulcers, ectopy, erythma and discharge. Patients with a positive urine pregnancy test, recent antibiotic therapy, yeast infection and genital tuberculosis were excluded from the study. The participants were briefed that their blood and vaginal swab samples will be used for research purposes, and written consent was obtained. This study has been approved by the University of Malaya Medical Centre Medical Ethics Committee.

### DNA extraction

Endocervical swabs were collected by using a Floqswab (Copan, Brescia, Italy) and transferred to a laboratory in a UTM-RT universal transport medium tube (Copan). The samples were vortex mixed and the cells were centrifuged at 400× g for 10 min. The cells recovered were lysed and separated with phenol chloroform. The DNA was precipitated using 1:10 volume of 3 M sodium acetate and isopropanol at −20 °C overnight incubation. The samples were washed and eluted with TE buffer.

### PCR amplification

Diagnosis of *C. trachomatis* was performed as described [15]. Nested PCR was performed to amplify the genes that encode for Major Outer Membrane Protein (MOMP) and plasmid using outer and inner primers (Table 4). PCR samples were run in a Veriti Thermal Cycler (Applied Biosystems, Essex, UK) at the following conditions: 95 °C for 3 min, 30 cycles of 95 °C for 30 s, 50 °C for 1 min, and 72 °C for 1 min, followed by a final extension at 72 °C for 7 min using HelixAmp Taq DNA polymerase (Nanohelix, Daejeon, South Korea). The first round PCR was amplified using 50 ng of DNA isolated from endocervical swabs with outer primer pairs. Then, 1 μl of first round PCR product was used as the template in the second round PCR and amplified with inner primer pairs. The amplicons were run on gel electrophoresis using 1.5 % agarose gel and viewed under a UV transilluminator. Human β-globin gene was used as a positive control to confirm successful DNA retrieval from patient swabs. PCR amplification for human β-globin primer was run separately in PCR master mix containing a total volume 50 μl as follows: 1 μl template DNA, 200 mM of each of dNTP, 25 pmol of each primer, 4 mM Tune Up solution, 5 μl of PCR buffer and 2.5 units of HelixAmp Taq DNA polymerase recombinant (Nanohelix, Daejeon, South Korea). PCR samples were run at the following conditions: 95 °C for 3 min, 30 cycles amplification consisted of 95 °C for 30 s, 50 °C for 1 min, and extension at 72 °C for 1 min, followed by final extension at 72 °C for 7 min.

**Table 4:** PCR primers used in *C. trachomatis* conventional PCR and real-time PCR diagnosis. For nested PCR amplification of *Momp* and plasmid, outer primer pairs were used at first round PCR, while inner primer pairs were used at second round PCR amplification. For real-time PCR diagnosis, *Momp* inner primer pairs, plasmid *Pgp8* inner primer pairs and plasmid *Pgp1* primer pairs were used

Target genes	Primer	Sequence (5′-3′)	Amplicon size
*Momp*	Outer forward	TTGTTTTCGACCGTGTTTTG	455 bp
	Outer reverse	AGCRTATTGGAAAGAAGCBCCTAA	
	Inner forward	AAACWGATGTGAATAAAGARTT	395 bp
	Inner reverse	TCCCASARAGCTGCDCGAGC	
Plasmid *Pgp8*	Outer forward	TTGGCYGCTAGAAAAGGCGATT	212 bp
	Outer reverse	TCCGGAACAYATGATGCGAAGT	
	Inner forward	AACCAAGGTCGATGTGATAG	150 bp
	Inner reverse	TCAGATAATTGGCGATTCTT	
Plasmid *Pgp1*	Forward	TTCTTTGATGGCTTCCCAAC	456 bp
	Reverse	ACGATTTTCTCCAACCGATG	
*β-globin*	Forward	GAAGAGCCAAGGACAGGTAC	268 bp
	Reverse	CAACTTCATCCACGTTCACC	

### Quantitative real-time PCR amplification

A quantitative real-time PCR assay was used to confirm the diagnosis and to determine the plasmid copy numbers in patient samples. DNA samples isolated from the vaginal swabs of *C. trachomatis*-infected patients were prepared. Mastermix containing 1 μl template DNA, 1× SsoAdvanced Universal SYBR Green Supermix (Biorad, Hercules, CA), 10 pmol primers were run using a Mx3000 Stratagene thermacycler (Agilent Technologies, Santa Clara, CA). Primers used were inner primers for *Momp*, inner primer for plasmid *Pgp8*, and plasmid *Pgp1* primers. Plasmid copy number for each patient was calculated as below:

$$ Plasmid\; copy\; number=\frac{Average\left({C}_TPgp1+{C}_TPgp8\right)}{C_T MOMP} $$

### Sequencing

Amplification of an approximately 871 bp fragment of *OmpA* was performed by nested PCR. The first PCR step was carried out with outer primer pair (Table 1) using 10 μl of DNA extracted from swabs. Amplification was performed in a final reaction volume of 50 μl containing 0.3 μM of each primer, 0.2 mM of dNTPs, 3 μl of TuneUp solution (Nanohelix, Korea) and 5U of HelixAmp™*Taq* DNA polymerase (Nanohelix, Korea). The first amplification conditions consisted of initial polymerase activation at 94 °C for 2 min; 40 cycles of 94 °C for 45 s, 60 °C for 45 s and 72 °C for 90 s and a final elongation step at 72 °C for 5 min. In the second round PCR, 3 μl of product from the first PCR step was amplified using inner primer pairs (Table 1). Nested PCR conditions consisted of 95 °C for 5 min; 40 cycles of 94 °C for 1 min, 60 °C for 1 min and 72 °C for 2 min and a final elongation step at 72 °C for 10 min. The amplified products were visualized by electrophoresis on 1.5 % agarose gel stained with GelRed solution (Biotium).

The 871 bp *OmpA* fragments obtained were purified using a QIAquick Gel Extraction kit (Qiagen) and processed using a BigDye® Terminator v3.1 Cycle Sequencing Kit (Applied Biosystems, Foster City, CA). The reaction mixtures were loaded onto a 3730xL DNA Analyzers (Applied Biosystems). The primers used for sequencing were inner primer pairs. Nucleotide sequence data were assembled by Bioedit and residues corresponding to flanking primers were excluded from analysis. Sequences were submitted to the standard nucleotide BLAST search engine at the National Center for Biotechnology Information (blast.ncbi.nlm.nih.gov/Blast.cgi) to determine the genotype.

### Statistical analysis

Statistical analysis was done using GraphPad PRISM version 5. For normally distributed data, an unpaired *t*-test was used. For categorical data (clinical symptoms), Fisher’s exact test was used, with a no-symptom group as control. Odd ratio (OR) and 95 % confidence interval (CI) were calculated. Statistical significance was determined at *P* < 0.05^*^, *P* < 0.01^**^ and *P* < 0.001^***^.

## Availability of data and materials

All data and materials are available upon request.

## Abbreviations

MOMP: Major outer membrane protein
OD: optical density
PCOS: polycystic ovary syndrome
PID: pelvic inflammatory disease
STD: sexually transmitted disease
