# Erratum: prevalence of plasmid-bearing and plasmid-free chlamydia trachomatis infection among women who visited obstetrics and gynecology clinics in Malaysia

**DOI:** 10.1186/s12866-016-0701-z

**Published:** 2016-05-23

**Authors:** Tee Cian Yeow, Won Fen Wong, Negar Shafiei Sabet, Sofiah Sulaiman, Fatemeh Shahhosseini, Grace Min Yi Tan, Elaheh Movahed, Chung Yeng Looi, Esaki M. Shankar, Rishein Gupta, Bernard P. Arulanandam, Jamiyah Hassan, Sazaly Abu Bakar

**Affiliations:** Department of Medical Microbiology, Tropical Infectious Disease Research and Education Center, Faculty of Medicine, University of Malaya, Kuala Lumpur, 50603 Malaysia; Faculty of Medicine, SEGi University, Petaling Jaya, 47810 Malaysia; Department of Obstetrics and Gynecology, Faculty of medicine, University of Malaya, Kuala Lumpur, 50603 Malaysia; Department of Pharmacology, Faculty of Medicine, University of Malaya, Kuala Lumpur, 50603 Malaysia; Center of Excellence in Infection Genomics, South Texas Center For Emerging Infectious Diseases, University of Texas at San Antonio, San Antonio, TX 78249 USA

## Erratum

The original version of this article [1] unfortunately contained a mistake in the author list and corresponding author details. The author Dr Rishein Gupta was incorrectly cited as “Rishien Gupta”. In addition Dr Negar Shafiei Sabet is now included as a corresponding author.

## References

[1] Yeow et al. BMC Microbiology (2016) 16:45 1186/s12866-016-0671-1

